# Oncological Safety of MRI-Informed Biopsy Decision-Making in Men With Suspected Prostate Cancer

**DOI:** 10.1001/jamaoncol.2024.5497

**Published:** 2024-12-12

**Authors:** Charlie A. Hamm, Patrick Asbach, Anna Pöhlmann, Ivo G. Schoots, Veeru Kasivisvanathan, Thomas O. Henkel, Manfred Johannsen, Thomas Speck, Alexander D. J. Baur, Matthias Haas, Federico Collettini, Tobias Penzkofer, Lynn J. Savic, Frank Konietschke, Lothar Weißbach, Bernd Hamm, Frank König, Hannes Cash

**Affiliations:** 1 Department of Radiology, Charité–Universitätsmedizin Berlin, Corporate Member of Freie Universität Berlin and Humboldt-Universität zu Berlin, Berlin, Germany; 2 Berlin Institute of Health (BIH), Berlin, Germany; 3 Institute of Biometry and Clinical Epidemiology, Charité–Universitätsmedizin Berlin, Corporate Member of Freie Universität Berlin and Humboldt-Universität zu Berlin, Berlin, Germany; 4 Department of Radiology and Nuclear Medicine, Erasmus University Medical Center, Rotterdam, the Netherlands; 5 Department of Radiology, Netherlands Cancer Institute, Amsterdam, the Netherlands; 6 Division of Surgery and Interventional Science, University College London, London, England, United Kingdom; 7 German Society of Urology (DGU), Berlin, Germany; 8 Working Group of Focal and Microtherapy of the German Society of Urology (DGU), Berlin, Germany; 9 Urology Practice Ihre Urologen Berlin MVZ GmbH, Berlin, Germany; 10 Urology Practice Johannsen & Laux, Berlin, Germany; 11 Urology Practice Speck, Berlin, Germany; 12 Gesundheitsforschung für Männer gGmbH, Berlin, Germany; 13 Department of Urology, University Hospital Magdeburg, Magdeburg, Sachsen-Anhalt, Germany

## Abstract

**Importance:**

The magnetic resonance imaging (MRI) pathway for diagnosing clinically significant prostate cancer (csPCa; defined as International Society of Urological Pathology grade group ≥2) uses multiparametric MRI (mpMRI) for prostate biopsy (PB) decision-making. However, the intermediate impact on patient outcomes in men with negative MRI results avoiding PB and men with positive MRI results without PCa remains unknown.

**Objective:**

To assess the feasibility and safety of a community-based MRI diagnostic strategy in men with suspected PCa using 3-year active monitoring.

**Design, Setting, and Participants:**

This multisite, longitudinal cohort trial took place across 54 community-based urology practices and 2 radiology imaging centers at a referral academic institution in Berlin, Germany. Eligible participants aged 18 to 75 years with clinically suspected PCa were enrolled between September 2016 and December 2017 and monitored for 3 years. Final analysis was reported on December 23, 2023.

**Exposures:**

Participants underwent 3-T mpMRI. Men with findings suspected to be PCa were recommended for targeted PB (diagnostic phase). Men with negative mpMRI results or positive mpMRI results with benign findings at PB were systematically monitored for 3 years (monitoring phase). Clinical visits were recommended every 6 months.

**Main Outcomes and Measures:**

The total proportion of men avoiding PB and those with csPCa.

**Results:**

A total of 593 men (median [IQR] age, 64 [58-70] years) underwent mpMRI, with 286 (48%) having negative MRI results, 261 (44%) avoiding PB initially, and 242 (41%) avoiding PB over 3 years. csPCa was detected in 161 (27%) men after immediate PB, increasing to 172 (29%) men after 3 years. Seven men with negative MRI results were diagnosed with PCa by immediate PB (including 4 cases of csPCa), while 279 entered monitoring. Three-year monitoring was completed by 233 (84%) men, with 7 diagnoses of csPCa. Of 307 men with positive MRI results, 58 (19%) showed no PCa after immediate PB, of which 41 (71%) completed monitoring and 4 (7%) were diagnosed with csPCa.

**Conclusions and Relevance:**

In this cohort study, men with negative mpMRI results avoiding biopsy were not at elevated risk of csPCa. The study confirms the oncological safety of the prebiopsy MRI strategy of avoiding an immediate PB after negative MRI results when a programmatic safety net is in place.

## Key Points

**Question:**

Is it safe to omit biopsy following negative magnetic resonance imaging (MRI) results in men with clinically suspected prostate cancer?

**Findings:**

In this cohort study involving 593 biopsy-naive men, 48% had negative MRI results, 86% of whom avoided biopsy over 3 years. After 3 years of programmatic monitoring, clinically significant prostate cancer was detected in 4% of men with negative MRI results who exhibited an ongoing elevated clinical risk.

**Meaning:**

This study demonstrates the high negative predictive value of prebiopsy MRI, showing that men with negative MRI results may not be at an elevated risk for prostate cancer and can safely avoid biopsy when appropriate monitoring measures are implemented.

## Teaser

This cohort study assesses the feasibility and safety of a community-based magnetic resonance imaging (MRI) diagnostic strategy in men with suspected prostate cancer using 3-year active monitoring.

## Introduction

Prostate cancer (PCa) is the second most common cancer and fifth leading cause of cancer-related deaths in men worldwide.^1^ Therefore, practitioners in ambulatory care see a large number of patients with suspected PCa on a daily basis, making the management of these patients of substantial health economic importance. Men at risk of PCa undergo serum prostate-specific antigen (PSA) testing and digital rectal examination (DRE). The new diagnostic standard according to guideline recommendations entails prebiopsy magnetic resonance imaging (MRI) enabling the localization of index lesions in the prostate, thereby allowing for targeted prostate biopsies (PBs).^2^ This so-called MRI-directed biopsy pathway (MRI pathway) has demonstrated an improved detection rate of clinically significant PCa (csPCa), suggesting a paradigm shift for the diagnosis and management of PCa.^3,4,5^ In addition to improving the detection of csPCa in men with MRI targets, a critical advantage of the MRI pathway is that it can serve as a triage tool for biopsy.^2^ Specifically, data in a Cochrane meta-analysis indicated that approximately one-third of men can safely avoid a PB within the MRI pathway.^3^ Furthermore, a high negative predictive value of MRI (>90%) in biopsy-naive men at a prevalence of 30% significant disease has also been demonstrated in several meta-analyses.^3,6^ However, most landmark trials that have led to the introduction of MRI as standard of care did not routinely include follow-up of men with negative MRI results or men with positive MRI results but negative biopsy results.^4,5,7^ Despite guideline recommendations, the oncological safety of using MRI as a triage tool for omitting biopsy still lacks prospective verification.^8^ Furthermore, an unproductive PB after positive MRI results poses a clinical dilemma during follow-up, with minimal prospective data available.^9^ This prospective, multisite, observational longitudinal cohort trial was designed to assess the feasibility, safety, and cancer detection rate of the prostate MRI pathway in a community-based setting using 3-year active monitoring.

## Methods

### Study Design

This prospective, multisite, longitudinal cohort trial is registered at the German Clinical Trials Register database (DRKS00010726), and the trial design has been published previously.^10^ Biopsy-naive men with suspected PCa were enrolled by 54 community-based urology practices and referred to undergo MRI at 1 of 2 radiology centers of a tertiary academic institution. Participants were categorized by MRI as having (1) negative MRI results with a low risk of PCa and (2) positive MRI results with an intermediate/high-risk of PCa, based on Prostate Imaging Reporting and Data System (PI-RADS) scoring.^2,11^ Men with negative MRI results (PI-RADS score 1-2) were recommended not to undergo PB but underwent biannual follow-up visits at the referring urology practice for 3 years (monitoring phase). The purpose of active monitoring was to define the number of PBs that could be safely avoided in men with negative MRI results. Men with positive MRI results (PI-RADS score 3-5) underwent systematic and targeted transrectal ultrasound (TRUS)–guided PB (diagnostic phase). Men with benign findings at biopsy (cancer negative) were also actively monitored for 3 years. The purpose was to define the PCa rate in men with positive MRI results.

The ethics committee at Charité–Universitätsmedizin Berlin, Corporate Member of Freie Universität Berlin and Humboldt-Universität zu Berlin in Berlin, Germany approved the study (EA1/019/16). All participants provided written informed consent. This study followed Strengthening the Reporting of Observational Studies in Epidemiology (STROBE) reporting guidelines.

### Participants

Men aged 18 to 75 years were eligible for enrollment between September 23, 2016, and December 11, 2017, if they had not undergone PB previously and had been referred with clinically suspected PCa based on an elevated serum PSA level, an abnormal DRE, or both. The PSA cutoffs were determined at the enrolling urologist’s discretion, with no upper limit specified. Exclusion criteria included suspected extraprostatic extension, suspected metastases, prior pelvic surgery, contraindications to multiparametric MRI (mpMRI), or participation in other interventional trials.^10^

### Procedures

#### mpMRI (Index Test)

mpMRI was performed with 3-T MRI scanners, including T2-weighted, diffusion-weighted, and dynamic contrast-enhanced sequences according to quality standards set by consensus guidelines (eTable 1 in Supplement 1).^11^ Images were interpreted in consensus by 2 of 4 radiologists (P.A., M.H., A.D.J.B., F.C.); in case of disagreement, an additional experienced radiologist (B.H.) was consulted (eAppendix in Supplement 1). The likelihood of having csPCa was scored according to the PI-RADS, version 2, scoring system.

#### Active Monitoring

Men with negative MRI results were monitored closely over a 3-year period with biannual visits at the enrolling urology practice. Monitoring included serum PSA, DRE, and TRUS according to clinical risk assessment by the treating urologist. The treating urologist was free to perform PB and/or follow-up (FU)-MRI at any time point during the trial, if clinically indicated. The same monitoring protocol was applied to men with positive MRI results but cancer-negative immediate PB. Three-year monitoring was considered complete if the last visit was more than 33 months and/or last serum PSA test was obtained more than 30 months after baseline MRI.

#### PB

Men with positive MRI results underwent TRUS-guided targeted and systematic PB using real-time ultrasonographic guidance. Biopsies were performed by the urologist enrolling the participant in the study using a previously reported 10- to 12-core sampling pattern following standard of care.^10,12^ MRI-targeted PB was performed either cognitively or software assisted (MRI–ultrasonographic fusion), based on the urologist’s discretion. In men with a PI-RADS score of 4 or higher at baseline MRI and a cancer-negative cognitive biopsy, software-assisted fusion rebiopsy was advised.

### Outcomes

This trial aimed to evaluate the feasibility and safety of the MRI pathway in a community setting using 3-year active monitoring. Primary outcomes were (1) the proportion of PBs avoided using prebiopsy MRI, (2) the proportion of men with clinically insignificant PCa (iPCa) by International Society of Urological Pathology grade group (GG) 1, and (3) the proportion of men with csPCa (GG ≥2) detected in men with negative MRI results (low risk) and positive MRI results (intermediate/high risk). Results from systematic PB in men at low risk and results from systematic and targeted PB in men with intermediate/high risk were used for the primary outcome measure. Exclusion of csPCa was determined after 3 years in both study groups, defining men with no PB or cancer-negative PBs as having no cancer.

### Statistical Analysis

The preemptively performed sample-size calculation was published previously.^10,13^ Medians and IQRs were used to describe continuous variables at baseline and during monitoring. Cumulative incidence of PB, FU-MRI, and PCa diagnosis was assessed using Aalen-Johansen estimators with the survfit function from the R survival package.^14^ All data were analyzed with the statistical software package R, version 4.2.2 (R Project for Statistical Computing), using core and survival packages. Final analysis was reported on December 23, 2023.

### Quality Control

Data were gathered by licensed trial monitors. On completion of the 3-year monitoring phase, data were reviewed for transcription errors by an independent data and safety monitoring committee. Data were checked for consistency with the regional cancer registry for active participants with either inconclusive data or less than 1 follow-up visit per year.

## Results

### Trial Population

**Figure 1.**
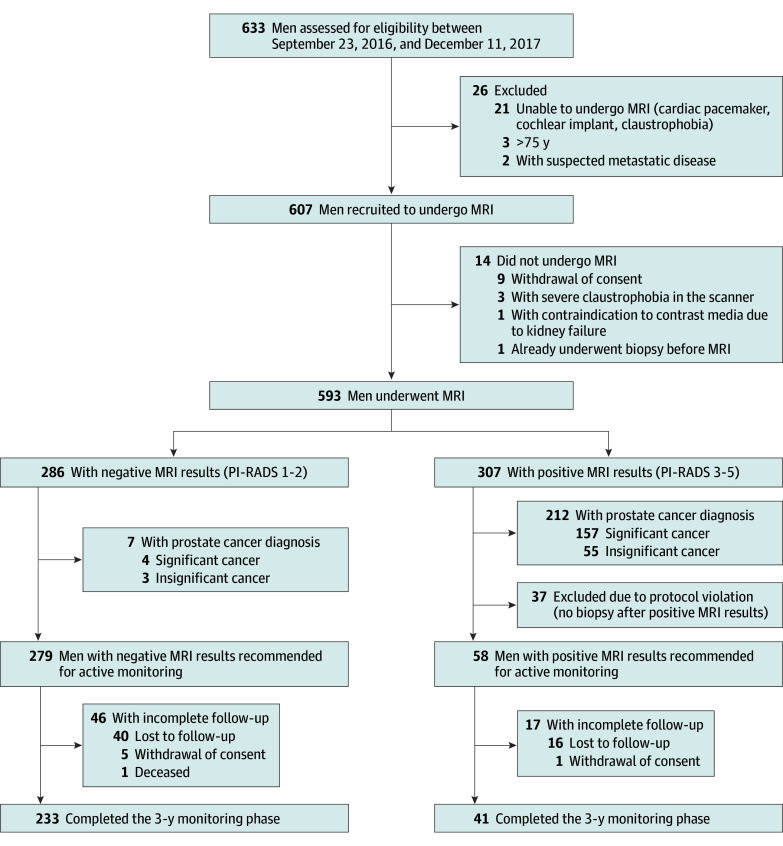
Trial Profile Insignificant cancer was defined as International Society of Urological Pathology grade group 1 and significant cancer as grade group 2 or higher. MRI indicates magnetic resonance imaging; PI-RADS, Prostate Imaging Reporting and Data System score.

**Figure 2.**
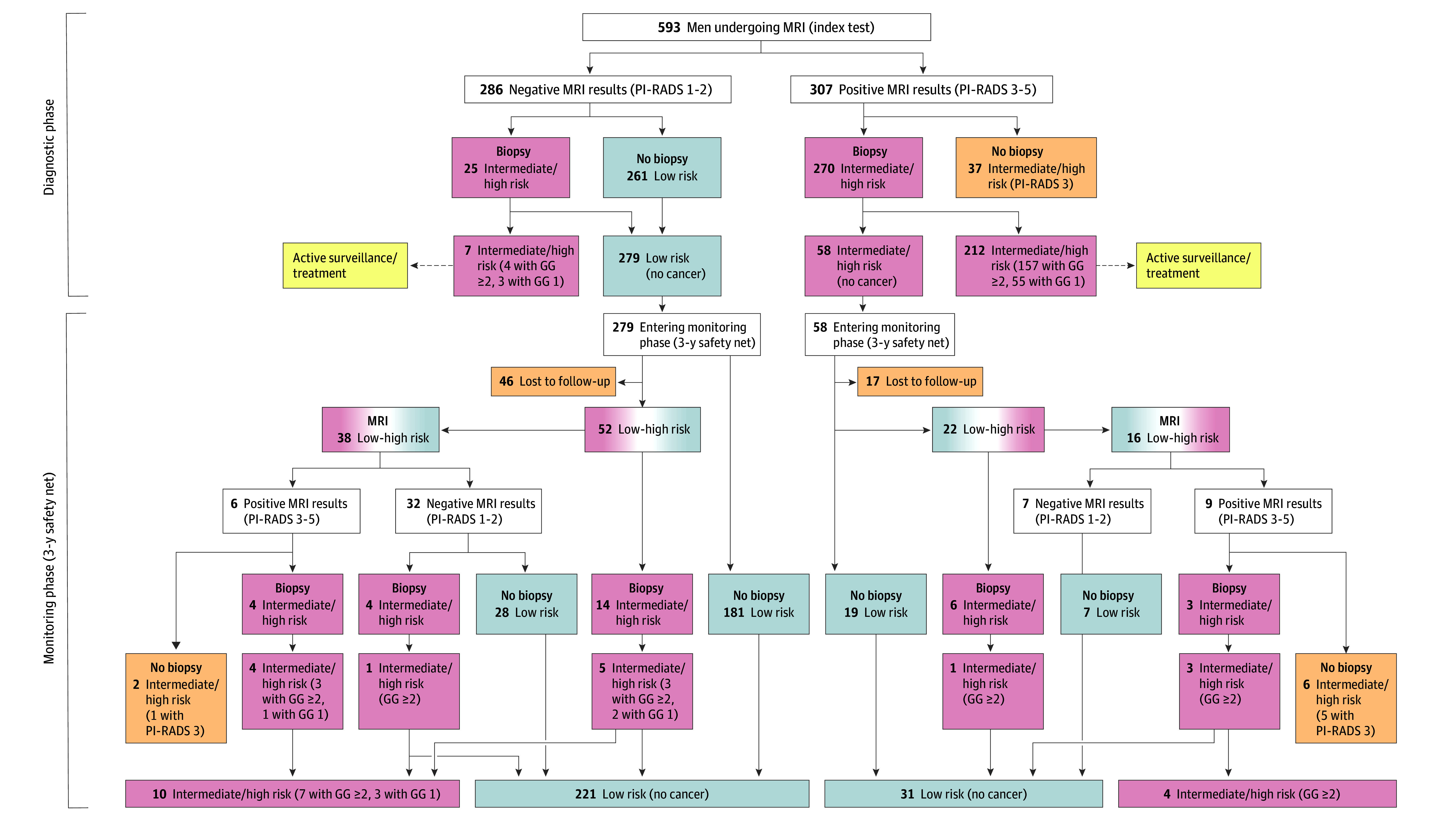
Magnetic Resonance Imaging (MRI)–Directed Pathway With Active Monitoring for 3 Years Prostate cancer (PCa) risk assessment was conducted at the discretion of the consulting urologist, considering a combination of factors such as MRI findings, serum prostate-specific antigen levels, clinical examination results, and biopsy outcomes. After MRI, 261 of 286 (91%) men with negative MRI results did not undergo biopsy in the diagnostic phase. Among the 25 men with negative MRI results who underwent immediate biopsy, 7 were diagnosed with PCa (4 with clinically significant PCa [csPCa], as defined by International Society of Urological Pathology grade group [GG] ≥2). Conversely, 279 men with negative MRI results entered the 3-year monitoring phase with a strict protocol in ambulatory setting, which was successfully completed by 233 (84%). Of the 279 men with negative MRI results, 221 (79%) were able to continue active monitoring for 3 years without an elevated risk of PCa. Of the 307 men with positive MRI results, 270 (88%) underwent immediate biopsy, revealing csPCa in 157 (58%). A majority of men not undergoing immediate biopsy showed a Prostate Imaging Reporting and Data System (PI-RADS) score of 3 (23 of 37 men). A total of 58 men with positive MRI results entered active monitoring after cancer-negative biopsy, 41 (71%) successfully completed the monitoring phase, and 4 (7%) were diagnosed with csPCa.

A total of 607 men were enrolled (median [IQR] per practice, 11 [6-18]; eTable 2 in Supplement 1), from which 593 men with a median (IQR) age of 64 (58-70) years underwent MRI (Table 1 and Figure 1). The cohort consisted of 307 (52%) men with positive MRI results and 286 (48%) men with negative MRI results (Table 2). In the diagnostic phase, 58 (10%) men had iPCa and 161 (27%) had csPCa (Figure 2). After a median (IQR) of 36 (34-37) months of monitoring, the iPCa prevalence remained stable at 10% (62 of 593 patients), while a slight increase to 29% was observed for csPCa (172 of 593 patients). Overall, 44 of the 286 (15%) men with negative MRI results underwent PB at some point during the study, while 242 of the total 593 (41%) did not undergo PB.

**Table 1.** Baseline Participant Characteristics

Characteristic	No. (%)		
	All (N = 593)	PI-RADS score	
		1-2 (n = 286)	3-5 (n = 307)
Age, median (IQR), y	64 (58-70)	63 (59-69)	64 (57-70)
PSA, median (IQR), ng/mL	5.8 (4.9-7.9)	5.9 (4.9-7.8)	5.8 (4.9-8.2)
Prostate volume, median (IQR), mL^a^	43 (31-60)	53 (39-69)	36 (26-48)
PSA density, median (IQR), ng/mL^2,^^b^	0.14 (0.10-0.20)	0.11 (0.08-0.16)	0.17 (0.12-0.24)
**Digital rectal examination**			
Normal findings	512 (86)	251 (88)	261 (85)
Abnormal findings	72 (12)	30 (10)	42 (14)
Not performed	9 (2)	5 (2)	4 (1)
**Transrectal ultrasound**			
Normal findings	435 (73)	206 (72)	229 (75)
Abnormal findings	58 (10)	19 (7)	39 (13)
Not performed	100 (17)	61 (21)	39 (13)

Abbreviations: PI-RADS, Prostate Imaging Reporting and Data System; PSA, prostate-specific antigen.

SI conversion factor: To convert PSA to micrograms per liter, multiply by 1.

^a^ Prostate volume at baseline magnetic resonance imaging using multiplanar segmentations.

^b^ PSA density was calculated using the PSA level at recruitment and prostate volume determined by segmentation on magnetic resonance imaging.

**Table 2.** Magnetic Resonance Imaging and Biopsy Results During the Diagnostic Phase

Characteristic	PI-RADS score, No. (%)^a^^,^^b^				
	1 or 2 (n = 286)	3 (n = 71)	4 (n = 151)	5 (n = 85)	Total (N = 593)
Clinically significant prostate cancer	4 (1)	8 (11)	81 (54)	68 (80)	161 (27)
GG 2	2 (50)	4 (50)	42(52)	23 (34)	71 (44)
GG 3	1 (25)	2 (25)	14 (17)	14 (21)	31 (19)
GG 4/5	1 (25)	2 (25)	25 (31)	31 (46)	59 (37)
Insignificant prostate cancer (GG 1)	3 (1)	9 (13)	35 (23)	11 (13)	58 (10)
No prostate cancer	18 (6)	31 (44)	25 (17)	2 (2)	76 (13)
No biopsy	261 (91)	23 (32)	10 (7)	4 (5)	298 (50)

Abbreviations: GG, International Society of Urological Pathology grade group; PI-RADS, Prostate Imaging Reporting and Data System.

^a^ Two men (PI-RADS score of 2 and 5) included herein were diagnosed with GG 2 prostate cancer at prostatectomy during the diagnostic phase, but no biopsy was documented.

^b^ Values may not sum to 100% due to rounding.

### Findings in Men With Negative MRI Results During the Diagnostic Phase

Of the 286 men with negative MRI results, 261 (91%) did not undergo immediate PB, representing 44% (261 of 593 patients) of the study cohort. Twenty-five (9%) men did undergo biopsy following negative MRI results (median [IQR] time after baseline MRI, 7 [4-9] months), showing 3 cases of iPCa and 4 of csPCa, demonstrating a negative predictive value for PCa and csPCa of 98% (95% CI, 96%-100% [279 of 286 patients]) and 99% (95% CI, 98%-100% [282 of 286 patients]), respectively (eTables 3-5 in Supplement 1). Subsequently, 7 (2%) men with negative MRI results having PCa did not enter the monitoring phase and were recommended to undergo treatment (Figure 2).

### Findings in Men With Positive MRI Results During the Diagnostic Phase

Of 307 men with positive MRI results, 270 (88%) underwent immediate PB (median [IQR] time after baseline MRI, 43 [29-74] days), while 37 (12%) did not and subsequently were excluded from further analysis due to protocol violation (23 of the 37 had a PI-RADS score of 3; eTable 6 in Supplement 1). Biopsy results revealed GG 1 or higher, 2 or higher, and 3 or higher PCa in 212 (69%), 157 (51%), and 88 (29%) men, respectively. In men with PI-RADS scores of 3, 4, and 5, GG 2 or higher was detected in 8 of 48 (17%), 81 of 141 (57%), and 68 of 81 (84%), respectively, while GG 3 or higher PCa was detected in 4 of 48 (8%), 39 of 141 (28%), and 45 of 81 (56%), respectively (eFigure 1 and eTable 7 in Supplement 1). A more in-depth analysis of biopsy results, considering the biopsy method and location, and radical prostatectomy concordance is presented eTables 8 through 10 and eFigure 2 in Supplement 1. Finally, 58 of 307 (19%) men with positive MRI results and cancer-negative PB results entered the monitoring phase.

### Outcome of Men With Negative MRI Results Undergoing Active Monitoring

**Figure 3.**
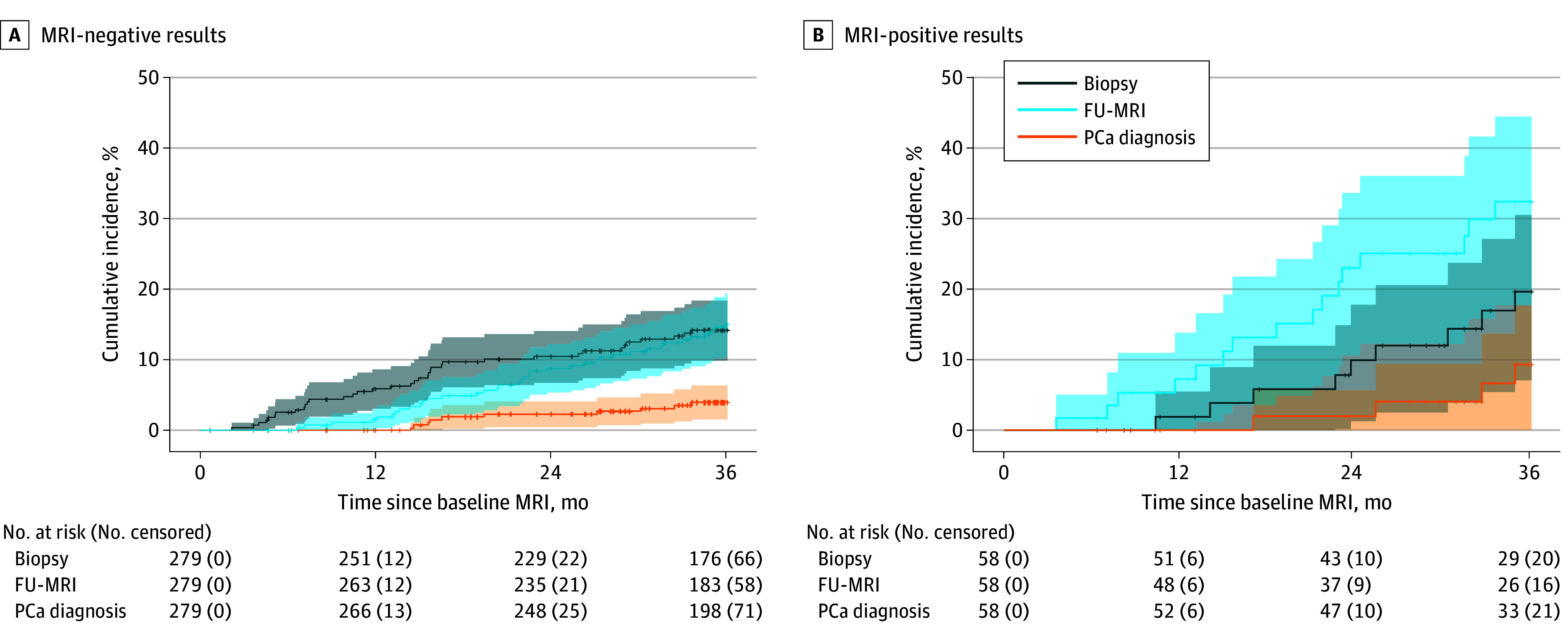
Cumulative Incidence of Events During 3 Years of Active Monitoring After Prostate Magnetic Resonance Imaging (MRI) Graphs show Kaplan-Meier estimates for 279 men with negative MRI results and 58 men with positive MRI results and cancer-negative biopsy who were actively monitored for 3 years with a strict protocol in an ambulatory setting. Data presented were calculated using number of events occurring within 36 months after baseline MRI. Shaded areas represent the 95% CIs, and vertical lines indicate censoring. At 36 months, the median (IQR) cumulative incidence for follow-up MRI (FU-MRI) scans and prostate biopsies in men with negative MRI results were 15.0% (10.4%-19.3%) and 14.2% (9.8%-18.3%), respectively, revealing prostate cancer (PCa) in 4.0% (1.5%-6.4%). Median (IQR) cumulative incidence of repeat biopsy and FU-MRI was substantially higher among men with positive MRI results, with 19.7% (7.1%-30.5%) and 32.4% (17.7%-44.5%), respectively; only 9.3% (<1%-17.7%) of men discontinued active monitoring to active treatment (including active surveillance) as a result of PCa detection (all with International Society of Urological Pathology grade group ≥2).

Three-year monitoring was completed by 233 of 279 (84%) men with negative MRI results, and the median (IQR) cumulative incidence of PB, FU-MRI, and PCa diagnosis at 3 years was 14.2% (9.8%-18.3%), 15.0% (10.4%-19.3%), and 4.0% (1.5%-6.4%), respectively (Figure 3). csPCa was detected in 7 of 279 (3%) men with negative MRI results (7 of 233 [3%] men with complete monitoring), including 4 with GG 2, 1 with GG 3, 1 with GG 4, and 1 with GG 5 PCa. Overall, MRI results showed a negative predictive value for csPCa of 96% (95% CI, 94%-98% [275 of 286 patients]) at 3 years.

Among the 233 men who completed the monitoring phase, clinical examinations and PSA testing were performed a median (IQR) of every 6 (5-7) months and 4 (3-6) months, respectively (eFigure 3 in Supplement 1). In terms of clinical examinations, DRE was performed in 229 (98%) men at regular intervals (median [IQR] of 3 [2-4] examinations at a 6 [6-12] month interval), with abnormal findings in 18 (8%). TRUS examination was performed in fewer men (175 of 233 [75%]) and less frequently (median [IQR] of 2 [1-4] examinations at a 6 [7-12] month interval), showing abnormal findings in 28 (12%). FU-MRI was performed in 38 (16%) men during monitoring, with 6 examinations showing new prostatic lesions (3 men with PI-RADS score of 3 and 4). Four of 6 men with positive FU-MRI results were confirmed to have csPCa, while biopsy was not performed in 2. Indicators for FU-MRI or PB during monitoring among 52 are detailed in the eAppendix in Supplement 1. Ten men underwent FU-MRI only after the end of the 3-year monitoring phase, and all but 1 (PI-RADS score of 4 with subsequently confirmed GG 2 csPCa) had negative results. Also, 1 patient with PCa was diagnosed just after 36 months of monitoring.

### Outcome of Men With Positive MRI Results Undergoing Active Monitoring

The 3-year monitoring was completed by 41 of 58 (71%) men with positive MRI results, and the median (IQR) cumulative incidence for a PB, FU-MRI, and PCa diagnosis at 3 years was 19.7% (7.1%-30.5%), 32.4% (17.7%-44.5%), and 9.3% (<1%-17.7%), respectively (Figure 3). csPCa was diagnosed in 4 of 58 (7%) men during monitoring (in 4 of 41 [10%] with complete monitoring), including 2 with GG 2, 1 with GG 3, and 1 with GG 4 PCa at a median (IQR) of 33 (26-34) months after cancer-negative PB result.

Among the 41 men who completed the monitoring phase, clinical examination and PSA testing were performed a median (IQR) of every 6 (5-7) months and every 4 (3-6) months, respectively (eFigure 4 in Supplement 1). In terms of clinical examinations, DRE was performed in 38 (93%) men at regular intervals (median [IQR] of 4 [3-5] examinations at a 7 [6-12] month interval), with abnormal findings in 3 (7%). TRUS examination was performed with similar frequency (median [IQR] of 3 [2-4] examinations at a 7 [6-12] month interval) in 34 (83%) men, with abnormal results in 9 (22%). FU-MRI results were negative in 7 of 16 men (44%; PI-RADS score of 2), while positive MRI results showed 2 upgradings (PI-RADS score of 4 to 5) and 3 downgradings (PI-RADS score of 4 to 3). Three of 9 men with positive FU-MRI results underwent biopsy, all showing csPCa. Six PBs were performed without FU-MRI, revealing 1 case of GG 2 PCa.

## Discussion

The main finding of this prospective, multisite trial demonstrated that prebiopsy prostate MRI, as an integral component of the MRI pathway, is feasible in a community-based setting and oncologically safe. Results showed a high negative predictive value (96%) for csPCa at a prevalence of 29%, potentially sparing men with negative MRI results from undergoing PB. Specifically, the MRI pathway prevented PB in 41% of all men and 86% of men with negative MRI results over 3 years, while csPCa was detected in only 4% of men with negative MRI results. Thus, prebiopsy MRI may not only improve cancer detection at biopsy, but also identify men who can safely omit biopsy. Additionally, both patients and urologists showed a high adherence to the safety net, providing initial evidence for the feasibility of a post-MRI safety net strategy in an everyday setting.

Based on prebiopsy MRI, 48% of men were advised not to undergo PB, matching results from recent prospective studies at academic centers (49% in the 4M trial^15^ and 55% in the MR-PROPER trial^16^). In comparison, pivotal studies establishing the clinical validity of prostate MRI, including a guideline-defining Cochrane meta-analysis,^3^ reported the potential of avoiding PBs in approximately 25% of men (PROMIS, 27%^5^; PRECISION, 28%^4^; and MRI-FIRST, 21%^7^). Notably, in studies with low prevalence of negative MRI results, a relatively high proportion of equivocal MRI findings has been reported (24%-28%^4,5,7^), while the low number of equivocal MRI findings (12%) and prevalence of negative MRI findings in this study are more in line with reporting standards of recent academic multicenter studies.^15,17^ However, biopsies were mainly performed in a decentralized setting by 54 office-based urologists, which marks a major difference from biopsy approaches in previous prospective trials. Nonetheless, the present detection rate of 27% for csPCa at PB during the diagnostic phase was comparable to rates of 25% to 39% in 4 large multicenter trials.^4,5,7,15^ Specifically, 58% of men with positive MRI results revealed csPCa, matching the 52% detection rate for targeted biopsies only in the MR-PROPER trial.^16^ Thus, MRI and biopsy findings in this trial align with the latest prospective study observations, making these findings during monitoring and the 3-year outcome likely to represent clinical practice across many institutions.

Using rigorous active monitoring over 3 years in a community-based care setting, we were able to demonstrate a 96% negative predictive value of MRI in biopsy-naive men at a csPCa prevalence of 29%. These findings provide prospective evidence for the negative predictive value of MRI, surpassing the 90.8% reported in a recent systematic literature review.^6^ The review found wide institutional variation in MRI reporting and cancer prevalence, although included studies were mainly conducted at academic centers and most studies used TRUS-guided PB as reference standard, while no follow-up data for men with negative MRI results were acquired. After 3 years of active monitoring, 41% of the men included were still spared from PB, while the risk of missed cancers can be considered low, as the 3-year csPCa detection rate across the whole population was 29%, matching results of large prospective studies investigating the MRI pathway with systematic biopsy as the reference standard (24%-27% in MR-PROPER,^16^ 4M,^15^ or a large Dutch cohort study^18^). Moreover, reducing the number of PBs certainly results in a lower number of detected iPCa and subsequent overtreatment, as potentially missed MRI-invisible cancers are mainly insignificant (GG 1) or low-volume localized GG 2 PCa.^8^ The strong adherence of patients and urologists to the active monitoring approach (safety net strategy), which is in line with current UK safety net guidelines for the follow-up of men with negative MRI results (guidelines that did not exist at the initiation of the trial), coupled with the reduced biopsy rate, proves both the applicability and safety of the MRI pathway in common clinical practice.^19^

During monitoring, the cumulative incidence of FU-MRI was 15% in men with negative MRI results, showing that clinical monitoring did not lead to a great increase of follow-up imaging and, therefore, higher costs. In comparison, the number of mandated FU-MRIs in men with positive MRI results was substantially higher (32%), while csPCa prevalence was slightly higher (4% and 7% for men with negative and positive MRI results, respectively). This highlights the challenges of monitoring men with positive MRI results and cancer-negative targeted biopsy, and only limited and nonsystematic evidence exists on this patient collective to date.^9,20,21,22^ A programmatic monitoring protocol, as demonstrated herein, can provide safety for men with persistent suspected PCa after prior MRI, as csPCa prevalence in both groups was below the overall European Association of Urology’s accepted risk threshold of 9% for csPCa.^23,24^ However, the present findings suggest the benefit of a standardized monitoring protocol for men with positive MRI results but negative biopsy results, showing a slightly elevated csPCa risk.^21,25^ On the contrary, a more individualized and patient-centered safety net may be suitable for men with negative MRI results. This is because these men do not exhibit an elevated risk for csPCa, similar to the cancer prevalence in a screening population.^26^

### Limitations

This study has certain limitations. First, all MRIs were performed at 2 radiology imaging centers of a high-volume academic institution with 2 expert radiologists reading the images in consensus, without using any computer-assisted diagnosis tools. While this may not reflect routine radiological reporting standards and, thus, limit the generalizability of the results, such collaboration could be envisioned to achieve high-quality MRI reports that maximize the benefits of the MRI pathway, particularly in a community-based setting. Moreover, the high detection accuracy achieved by office-based urologists using cognitive PBs highlights the necessity of expert-level MRI analysis. Second, in most men a cognitive biopsy approach was chosen by the urologists, whereas software-assisted fusion biopsy was rarely performed. The FUTURE trial with a similar high-quality MRI reading showed that both biopsy approaches had comparable csPCa detection rates.^27^ Third, the performance of the MRI pathway is prevalence dependent, and the study was conducted in a state with a high age-standardized PCa incidence rate (88.5 new cases per 100 000 inhabitants), compared to the Western European and high Human Development Index country averages.^1^ Additionally, negative predictive values are inherently dependent on the prevalence in the investigated population. While the true prevalence is unknown, we have estimated the csPCa prevalence for the present sample to be 29%. Lastly, patient risk stratification in this study was performed at the discretion of the treating urologist, without incorporating the latest advancements in risk-stratification techniques. The use of tools such as PSA density calculation or genetic testing could potentially further enhance patient safety and optimize patient management.^28,29,30^

## Conclusions

This cohort study validates the prostate MRI pathway by demonstrating that after expert readings, men with negative MRI results were not at an elevated risk for PCa and could safely avoid PBs in a community health care setting when a safety net was in place. By providing programmatic 3-year monitoring data for both patients with negative and positive MRI results, findings from this study address the gap of knowledge highlighted in current guidelines on the use of MRI in the diagnosis and management of PCa.

## Notes

Supplement 1.
eAppendix
**eTable 1.** Acquisition protocol and sequence parameters for multiparametric MR imaging **eTable 2.** Number of men included per community-based urology practice **eTable 3.** Men with prostate cancer after negative baseline MRI over 3 years **eTable 4.** Men undergoing prostate biopsy within the first year after negative baseline MRI **eTable 5.** Men undergoing prostate biopsy within three years after negative baseline MRI **eTable 6.** Men with positive baseline MRI but no immediate biopsy within the diagnostic phase **eTable 7. **Men with a PI-RADS 4 score at baseline MRI who were not found to have prostate cancer at initial biopsy **eTable 8.** Biopsy results over three years in regard to pre-biopsy MRI results for community-based practices and expert centers **eTable 9.** Agreement in Grade Groups between ultrasound-guided biopsies and prostatectomy throughout the study **eTable 10.** Adverse events **eFigure 1.** Prostate cancer detection rates of prostate biopsies by PI-RADS categories for no cancer, clinically insignificant and significant cancer **eFigure 2.** Association between MRI score and histology in ultrasound-guided biopsies and prostatectomy specimens **eFigure 3.** Average time interval between serum PSA tests during active monitoring of MRI-negative men **eFigure 4.** Average time interval between serum PSA tests during active monitoring of MRI-positive men

Supplement 2. Data Sharing Statement
