# Supplementary material for: Oncological Safety of MRI-Informed Biopsy Decision-Making in Men With Suspected Prostate Cancer
Source: JAMA Oncol. 2024 Dec 12;11(2):145–53. doi: 10.1001/jamaoncol.2024.5497 (PMC11843366; doi:10.1001/jamaoncol.2024.5497)
Supplement: Supplement 1. — eAppendix eTable 1. Acquisition protocol and sequence parameters for multiparametric MR imaging eTable 2. Number of men included per community-based urology practice eTable 3. Men with prostate cancer after negative baseline MRI over 3 years eTable 4. Men undergoing prostate biopsy within the first year after negative baseline MRI eTable 5. Men undergoing prostate biopsy within three years after negative baseline MRI eTable 6. Men with positive baseline MRI but no immediate biopsy within the diagnostic phase eTable 7. Men with a PI-RADS 4 score at baseline MRI who were not found to have prostate cancer at initial biopsy eTable 8. Biopsy results over three years in regard to pre-biopsy MRI results for community-based practices and expert centers eTable 9. Agreement in Grade Groups between ultrasound-guided biopsies and prostatectomy throughout the study eTable 10. Adverse events eFigure 1. Prostate cancer detection rates of prostate biopsies by PI-RADS categories for no cancer, clinically insignificant and significant cancer eFigure 2. Association between MRI score and histology in ultrasound-guided biopsies and prostatectomy specimens eFigure 3. Average time interval between serum PSA tests during active monitoring of MRI-negative men eFigure 4. Average time interval between serum PSA tests during active monitoring of MRI-positive men [file jamaoncol-e245497-s001.pdf]

Supplemental Online Content

Hamm CA, Asbach P, Pöhlmann A, et al. Oncological safety of MRI-informed biopsy decision-making in men with suspected prostate cancer. *JAMA Oncol*. Published online December 12, 2024. doi:10.1001/jamaoncol.2024.5497

eAppendix
eTable 1. Acquisition protocol and sequence parameters for multiparametric MR imaging
eTable 2. Number of men included per community-based urology practice
eTable 3. Men with prostate cancer after negative baseline MRI over 3 years
eTable 4. Men undergoing prostate biopsy within the first year after negative baseline MRI
eTable 5. Men undergoing prostate biopsy within three years after negative baseline MRI
eTable 6. Men with positive baseline MRI but no immediate biopsy within the diagnostic phase
eTable 7. Men with a PI-RADS 4 score at baseline MRI who were not found to have prostate cancer at initial biopsy
eTable 8. Biopsy results over three years in regard to pre-biopsy MRI results for community-based practices and expert centers
eTable 9. Agreement in Grade Groups between ultrasound-guided biopsies and prostatectomy throughout the study
eTable 10. Adverse events
eFigure 1. Prostate cancer detection rates of prostate biopsies by PI-RADS categories for no cancer, clinically insignificant and significant cancer
eFigure 2. Association between MRI score and histology in ultrasound-guided biopsies and prostatectomy specimens
eFigure 3. Average time interval between serum PSA tests during active monitoring of MRI-negative men
eFigure 4. Average time interval between serum PSA tests during active monitoring of MRI-positive men

This supplemental material has been provided by the authors to give readers additional information about their work.

© 2024 Hamm CA et al. *JAMA Oncol.*

eAppendix

MRI consensus readings

Imaging was performed at the Department of Radiology at the Charité Campus Benjamin Franklin and Charité Campus Virchow. Images were interpreted in consensus by two out of four radiologists (Matthias Haas, Alexander D. J. Baur, and Federico Collettini with 5-8 years and Patrick Asbach with more than ≥15 years of experience in prostate MRI). In case of disagreement, an additional experienced radiologist was consulted (Bernd Hamm with ≥15 years of experience).

Prostate biopsy method, location and performance

Immediate biopsies, including re-biopsies per study protocol, after baseline MRI included 227 cognitive fusion, 63 software-assisted (MRI–ultrasonographic fusion) and 24 systematic TRUS-guided biopsies, of which 131 (58%), 26 (41%), and 2 (8%) detected International Society of Urological Pathology grade group ≥2 PCa, respectively. Over the three-year study period, 78% (286/365) of biopsies were performed by community-based urologists. On the contrary, only 22% (79/365) underwent prostate biopsy at an expert urological center. Specifically, 23% (18/79) of biopsies at expert centers were repeat biopsies performed during the 1st, 2nd and 3rd year after initial biopsy (8, 7, and 3 biopsies, respectively). All biopsies performed by expert centers were MRI–ultrasonographic fusion biopsies, while community-based practices performed only 4 software-assisted biopsies. The majority of biopsies were performed transrectally (329/365, 90%), with a minority transperineally (36/365, 10%).

Indicators for MRI or prostate biopsy during monitoring

The predominant indicator for follow-up MRI or prostate biopsy in MRI-negative men during monitoring (n=52 men) was an increase in serum PSA levels. The median PSA increase leading to subsequent intervention in 81% (42/52) of men was 47% (IQR: 27-83%).

In 11% (6/52) of men, follow-up intervention was recommended after abnormal DRE or TRUS findings, despite stable or even falling PSA levels, revealing 1 International Society of Urological Pathology grade group 2 PCa. For the remaining participants (8%; 4/52) undergoing FU-MRI or PB, no direct indication could be identified.

In MRI-positive men, the predominant indication for follow-up MRI or repeat biopsy during monitoring was an increase in serum PSA, with a PSA increase of 49% (IQR: 23-76%). In 2 men follow-up intervention was recommended after abnormal TRUS findings.

© 2024 Hamm CA et al. *JAMA Oncol.*

Supplementary Tables

eTable 1. Acquisition protocol and sequence parameters for multiparametric MR imaging

Sequence	Plane	FOV (mm)	Slice thickness (mm)	In plane resolution (mm)	Flip angle (°)	TR (ms)	TE (ms)	b-values s/mm² (averages)	PAT factor	Acquisition	comment
**T2 TSE**	axial	180*180	3 (no gap)	0.5*0.5	160	4040	116	N/A	2	2D	-
	coronal	200*200	3 (no gap)	0.6*0.6	160	4000	112	N/A	2	2D	-
**EPI-DWI**	axial	220*220	3 (no gap)	1.4*1.4	90	4400	58	0 (4)<br>50 (4)<br>500 (7)<br>1000 (8)	4	2D	Images with a b-value of 1400 as well as ADC maps were calculated based on acquired b-values
**T1 TSE**	axial	320*240	3 (no gap)	0.6*0.6	160	800	11	N/A	4	2D	-
**T1 GRE (TWIST)**	axial	260*260	3 (no gap)	1.4*1.4	12	5	2	N/A	2	3D	A standard dose of 0.1 mmol/kg Gadobutrol was administered with an injection rate of 3 ml/s. Temporal resolution was 5 s, image acquisition was continued for 300 s.

Note. - TSE= turbo spin echo; EPI= echo-planar imaging; DWI= diffusion weighted imaging; GRE= gradient echo; TWIST= Time-resolved angiography with interleaved stochastic trajectories; FOV= field of view; TR= repetition time; TE= echo time; PAT= parallel acquisition technique; ADC= apparent diffusion coefficient; N/A= not applicable. Adapted from Baur et al. A prospective study investigating the impact of multiparametric MRI in biopsy-naïve patients with clinically suspected prostate cancer: The PROKOMB study. Contemporary Clinical Trials 56 (2017) 46–51.

eTable 2. Number of men included per community-based urology practice

Practice (ID)	Participants (n)
1	30
2	12
3	29
4	27
5	7
6	24

© 2024 Hamm CA et al. *JAMA Oncol.*

7	12
8	11
9	11
10	21
12	13
13	12
14	27
15	30
16	18
17	25
18	6
19	18
22	6
23	9
24	6
26	6
27	25
28	6
29	18
30	24
31	14
32	6
33	6
34	5
35	12
36	4
37	3

© 2024 Hamm CA et al. *JAMA Oncol.*

38	5
39	2
40	5
41	5
42	12
43	4
44	12
45	5
46	4
47	6
48	4
49	10
50	12
51	6
52	6
53	9
54	17
**Total**	**607**

© 2024 Hamm CA et al. *JAMA Oncol.*

eTable 3. Men with prostate cancer after negative baseline MRI over 3 years

		Baseline MRI				1st Follow-up MRI			2nd Follow-up MRI			TRUS-guided prostate biopsy			
		date	PI-RADS	PSA	PSAD	date	PI-RADS	PSA	date	PI-RADS	PSA	date	GG	number of positive cores	PSA
**PCa diagnosis within 1st year**	1*	14.12.2016	2	5.35	0.10	-	-	-	-	-	-	03.02.2017	2	N/A	N/A
	2	04.02.2017	2	12.55	0.33	-	-	-	-	-	-	18.08.2017	1	4/12	13.55
	3	13.12.2016	2	9.18	0.23	-	-	-	-	-	-	10.08.2017	3	2/12	5.2
	4	01.04.2017	2	4.38	0.13	-	-	-	-	-	-	20.12.2017	1	1/12	7.57
	5	24.03.2017	2	3.9	0.09	-	-	-	-	-	-	17.07.2017	2	1/12	3.6
	6	20.03.2017	2	11.92	0.19	-	-	-	-	-	-	23.05.2017	1	1/12	6
	7	07.06.2017	2	5.86	0.03	-	-	-	-	-	-	13.07.2017	4	3/20	5.86
**PCa diagnosis within 2nd year**	8	18.11.2016	2	7.42	0.21	-	-	-	-	-	-	06.03.2018	5	13/14	11.34
	9	28.10.2016	2	2.52	0.11	-	-	-	-	-	-	25.01.2018	2	4/12	2.86
	10	14.01.2017	2	3.75	0.10	-	-	-	-	-	-	21.02.2018	1	1/12	5.98
	11	18.11.2016	2	5	0.20	-	-	-	-	-		19.02.2018	2	5/12	8.04
	12	05.12.2016	2	5.3	0.14	-	-	-	-	-	-	18.01.2018	1	1/12	5.49
	13	22.04.2017	2	4.3	0.17	19.10.2017	2	5.48	11.10.2018	3	5.42	08.11.2018	2	5/14	5.42
**PCa diagnosis within 3rd year**	14	06.05.2017	2	4.38	0.16	09.08.2019	4	6.2	-	-	-	04.10.2019	1	2/12	6.57
	15	09.02.2017	2	7.59	0.27	17.07.2019	4	11.8	-	-	-	09.08.2019	3	8/11	11.8
	16	04.02.2017	2	5.36	0.21	18.04.2018	3	9	-	-	-	16.04.2019	4	2/11	14.1
	17	01.04.2017	2	5.73	0.26	14.12.2018	2	7.62	-	-	-	03.12.2019	2	1/10	9.38
**PCa diagnosis after 3rd year**	18	13.11.2016	2	7.75	0.15	-	-	-	-	-	-	18.11.2019	1	2/12	11.03
	19	10.12.2016	2	5.9	0.15	25.09.2019	2	11.02	02.12.2020	4	15.8	03.03.2021	2	7/10	N/A

Note. – MRIs were scored using the PI-RADS version 2 for the baseline MRI, while follow-up MRIs performed after October 2019 are reported using PI-RADS v2.1. Biopsy results are reported using the International Society of Urological Pathology grade group (GG) (the reported GG5 case was a Gleason score 9 cancer) and the ratio of positive biopsy cores (positive cores/total cores).*= Participant with no reported biopsy but prostate cancer diagnosis on prostatectomy. PCa= prostate cancer. PSA= serum prostate-specific antigen level. PSAD= PSA-density. DRE= digital rectal examination. TRUS= transrectal ultrasound. GG= Grade group of prostate cancer. N/A= not available.

© 2024 Hamm CA et al. *JAMA Oncol.*

eTable 4. Men undergoing prostate biopsy within the first year after negative baseline MRI

Baseline MRI score		Baseline			Monitoring		Follow-up MRI score		Prostate Biopsy						
1	2	PSA (median; IQR)	No. of men with abnormal DRE	No. of men with abnormal TRUS	No. of men with abnormal DRE	No. of men with abnormal TRUS	2	4	months after index MRI (median; IQR)	PSA (median; IQR)	GG1	GG2	GG3	GG4	No cancer
1	23	9.1 (5.7-10.8)	3	0	4	3	4	1	7 (4-9)	9.2 (5.8 - 12.2)	3	1	1	1	18

Note. – Within the first year after negative MRI, 24 men underwent prostate biopsy. Additionally, one man underwent prostatectomy (revealing Grade Group 2 prostate cancer) without a documented prior biopsy; this case is not included in the table above. MRIs were scored using the PI-RADS version 2 for the baseline MRI, while follow-up MRIs performed after October 2019 are reported using PI-RADS v2.1. Biopsy results are reported using the International Society of Urological Pathology grade group (GG). PSA= serum prostate-specific antigen level. DRE= digital rectal examination. TRUS= transrectal ultrasound.

eTable 5. Men undergoing prostate biopsy within three years after negative baseline MRI

Baseline MRI score		Baseline			Monitoring		Follow-up MRI score			Prostate Biopsy							
1	2	PSA (median; IQR)	No. of men with abnormal DRE	No. of men with abnormal TRUS	No. of men with abnormal DRE	No. of men with abnormal TRUS	2	3	4	months after baseline MRI (median; IQR)	PSA (median; IQR)	GG1	GG2	GG3	GG4	GG5	No cancer
2	41	6.4 (4.5-9.1)	9	3	8	8	7	1	4	12 (7-23)	8.3 (5.6 - 11.3)	6	5	2	2	1	27

Note. – One man underwent prostatectomy within the first year after baseline MRI (revealing Grade Group 2 prostate cancer) without a documented prior biopsy; this case is not included in the table above. MRIs were scored using the PI-RADS version 2 for the baseline MRI, while follow-up MRIs performed after October 2019 are reported using PI-RADS v2.1. Biopsy results are reported using the International Society of Urological Pathology grade group (GG). PSA= serum prostate-specific antigen level. DRE= digital rectal examination. TRUS= transrectal ultrasound.

© 2024 Hamm CA et al. *JAMA Oncol.*

eTable 6. Men with positive baseline MRI but no immediate biopsy within the diagnostic phase

Baseline			Baseline MRI score			Follow-up MRI score			Prostate biopsy				
PSA (median; IQR)	No. of men with abnormal DRE	No. of men with abnormal TRUS	3	4	5	2	3	4	No cancer	GG2	GG4	GG5	months after index MRI (median; IQR)
5.7 (4.9-7.9)	4	6	23	10	4	5	2	4	3	1	2	1	16 (14.5-22)

Note. – MRIs were scored using the PI-RADS version 2 for the baseline MRI, while follow-up MRIs performed after October 2019 are reported using PI-RADS v2.1. Biopsy results are reported using the International Society of Urological Pathology grade group (GG). PSA= serum prostate-specific antigen level. DRE= digital rectal examination. TRUS= transrectal ultrasound.

eTable 7. Men with a PI-RADS 4 score at baseline MRI who were not found to have prostate cancer at initial biopsy

Baseline			Follow-up MRI score				Repeat prostate biopsy					
PSA (median; IQR)	No. of men with abnormal DRE	No. of men with abnormal TRUS	2	3	4	5	months after negative biopsy (median; IQR)	GG1	GG2	GG3	GG4	No cancer
5.7 (4.8-7.2)	5	4	3	4	7	2	14 (8-23)	6	2	2	5	2

Note. – 17 out of 36 men with PI-RADS 4 at baseline MRI and negative targeted biopsy underwent rebiopsy. All follow-up MRIs were reported using PI-RADS v2.1. Biopsy results are reported using the International Society of Urological Pathology grade group (GG). PSA= serum prostate-specific antigen level. DRE= digital rectal examination. TRUS= transrectal ultrasound. Neg.= no prostate cancer.

© 2024 Hamm CA et al. *JAMA Oncol.*

eTable 8. Biopsy results over three years in regard to pre-biopsy MRI results for community-based practices and expert centers

**Community-based practices**					
**Biopsy result**	**MRI score**				
	**PI-RADS 1/2** (n=41)	**PI-RADS 3** (n=47)	**PI-RADS 4** (n=126)	**PI-RADS 5** (n=72)	**Total** (n=286)
clinically significant prostate cancer (GG≥2)	8	9	73	56	146
GG 2	5	5	39	19	68
GG 3	1	2	15	11	29
GG 4/5	2	2	19	26	49
insignificant prostate cancer (GG1)	7	5	26	7	45
No cancer	26	33	27	9	95
**Expert centers**					
**Biopsy result**	**MRI score**				
	**PI-RADS 1/2** (n=3)	**PI-RADS 3** (n=13)	**PI-RADS 4** (n=45)	**PI-RADS 5** (n=18)	**Total** (n=79)
clinically significant prostate cancer (GG≥2)	-	2	18	13	33
GG 2	-	1	7	4	12
GG 3	-	-	2	3	5
GG 4/5	-	1	9	6	16
insignificant prostate cancer (GG1)	-	5	15	4	24
No cancer	3	6	12	1	22

Note. - 8 participants underwent prostate TRUS-guided biopsy in a community-based urological practice as well as an expert center and are therefore listed twice. Biopsy reports of 10 men were missing and therefore not here included. On average, urologists conducted 3 prostate biopsies during the study period (IQR: 1-7). GG= International Society of Urological Pathology grade group, PI-RADS= Prostate Imaging–Reporting and Data System.

© 2024 Hamm CA et al. *JAMA Oncol.*

eTable 9. Agreement in Grade Groups between ultrasound-guided biopsies and prostatectomy throughout the study

Ultrasound-guided biopsy	Prostatectomy histology					
	GG 1	GG 2	GG 3	GG 4	GG 5	**Total**
GG 1	8	15	1	-	-	**24**
GG 2	-	35	6	3	-	**44**
GG 3	-	11	10	-	-	**21**
GG 4	-	7	21	3	5	**36**
GG 5	-	2	3	1	6	**12**
No prostate cancer	-	3	1	1	2	**7**
**Total**	**8**	**73**	**42**	**8**	**13**	**144**

Note.- GG= International Society of Urological Pathology grade group. All seven men with no cancer documented during the official study interval had a positive baseline MRI and follow-up biopsy as detailed below:
3x GG2 on prostatectomy: GG2 (1x during second year, 1x during third year) and GG3 (1x during third year) on biopsy after PI-RADS 4 at baseline MRI
1x GG3 on prostatectomy: GG4 on biopsy in the second year after PI-RADS 4 at baseline MRI
1x GG4 on prostatectomy: GG4 on biopsy in the third year after PI-RADS 4 at baseline MRI
2x GG5 on prostatectomy: GG4 on biopsy in the second year after PI-RADS 4 at baseline MRI and GG2 in the second year after PI-RADS 5 at baseline MRI and initial negative biopsy

© 2024 Hamm CA et al. *JAMA Oncol.*

eTable 10. Adverse events

	Specification	Begin	End	Severity score	Required treatment	Treatment/intervention specification	Required inpatient care	Major complication	Patient outcome
**Not associated with study related interventions**									
1	Urinary retention	27.10.17	14.01.18	3	yes	Holmium Laser Enucleation of the Prostate (HoLEP)	yes	yes	recovered
2	Urinary retention	20.11.17	06.02.18	2	yes	Holmium Laser Enucleation of the Prostate (HoLEP)	no	yes	recovered
3	acute liver failure	N/A	N/A	N/A	N/A	N/A	N/A	N/A	N/A
4	Myeloma	19.05.17	04.07.17	2	yes	Biopsy	yes	yes	recovered
5	Brain tumor	27.06.19	04.07.19	3	yes	Palliative care in a hospice	yes	yes	deceased
6	Fracture of lower leg	01.06.17	01.12.18	2	no	N/A	N/A	yes	recovered
7	High-grade lymphoma	17.02.18	17.02.18	3	yes	Oncological work up	yes	yes	deceased
8	Soft tissue infection	31.05.17	21.06.17	2	yes	Debridement	no	no	recovered
9	Prostatic abscess	28.05.18	10.07.18	2	yes	antibiotics	yes	yes	recovered
10	Cerebral hemorrhage	19.11.17	19.11.17	3	no	N/A	N/A	yes	deceased
11	Exitus letalis due to internal condition	26.04.19	26.04.19	3	no	N/A	N/A	yes	deceased
12	Bronchial carcinoma	01.03.18	04.06.18	3	yes	Resection	yes	yes	deceased
13	Urinary retention	11.01.18	26.02.18	2	yes	Holmium Laser Enucleation of the Prostate (HoLEP)	yes	yes	recovered
14	Acute myocardial infarction	05.07.17	05.07.17	2	yes	Stent implantation	yes	yes	recovered
15	Exitus letalis	03.03.21	03.03.21	3	no	N/A		yes	deceased
**Associated with MRI**									
1	Tinnitus	13.02.17	N/A	3	yes	Consultation with an ear, nose and throat (ENT) specialist	no	yes	persisting

© 2024 Hamm CA et al. *JAMA Oncol.*

**Associated with prostate biopsy**									
1	Macrohematuria	31.01.17	01.02.17	1	yes	Medical monitoring	yes	yes	recovered
2	Pain/prostatitis	20.03.17	28.03.17	1	yes	Antibiotic and anti-inflammatory medication (Cefpodixim, Ibuprofen)	no	no	recovered
3	Acute urinary retention	28.03.17	03.04.17	2	yes	urinary catheterization	no	no	recovered
4	Urosepsis and lobar pneumonia	02.07.17	12.07.17	1	yes	Antibiotics (Ciproflox, Tazobactam)	yes	no	recovered
5	Macrohematuria	31.01.17	01.02.17	1	yes	Medical monitoring	yes	yes	recovered
6	Macrohematuria	06.09.19	07.09.19	1	yes	Medical monitoring	yes	yes	recovered
7	Acute urinary retention	24.02.17	25.02.17	3	yes	urinary catheterization	yes	yes	recovered
8	Urosepsis in acute prostatitis with delirium	22.04.17	26.04.17	3	yes	Antibiotics (Ciprofloxacin, Sulbactam, Tazobac, Ampicillin)	yes	yes	recovered
9	Immediate post-biopsy circulatory collapse leading to a fall	18.04.18	18.04.18	1	yes	Medical monitoring and wound care	no	no	recovered
10	Macrohematuria+ urinary bladder tamponade	23.08.17	26.08.17	2	yes	Lavage of the bladder	yes	yes	recovered
11	Prostatitis	19.09.17	24.09.17	2	yes	Antibiotics, urinary catheterization	yes	yes	recovered
12	Fever	10.01.18	12.01.18	2	yes	Medical monitoring	no	yes	recovered
13	Viral infection	17.11.17	24.11.17	2	yes	Antibiotics, Cefpodoxim	no	no	recovered
**Associated with prostatectomy**									
1	Incontinence	01.09.18	N/A	2	yes	Pelvic Floor Gymnastics	no	no	persisting
2	Incontinence	08.01.18	07.06.18	1	yes	Pelvic Floor Gymnastics	no	no	recovered
3	Lymphocele	07.11.17	16.12.17	2	yes	Drainage installation	yes	yes	recovered
4	Infected lymphocele	08.11.17	16.11.17	2	yes	Drainage installation	yes	yes	recovered
5	Pain and urinary tract infection	30.10.18	20.12.18	2	yes	Antibiotics	no	no	recovered

Note. – Severity score: 1 - mild, 2 - moderate, 3 - severe. N/A= not available.

© 2024 Hamm CA et al. *JAMA Oncol.*

Supplementary Figures

eFigure 1. Prostate cancer detection rates of prostate biopsies by PI-RADS categories for no cancer, clinically insignificant and significant cancer

MRI reporting showed an excellent positive predictive value for clinically significant prostate cancer (International Society of Urological Pathology grade group (GG) ≥2), with 84% of men with Prostate Imaging–Reporting and Data System (PI-RADS) score 5 being subsequently diagnosed with GG≥2 cancer. In contrast, only 17% of individuals with PI-RADS 3 (12% of the entire cohort with PI-RADS 3) were found to have GG≥2 cancer, suggesting that a substantial proportion (83%) of individuals underwent unnecessary biopsies, considering the detection of GG1 cancer as overdiagnosis. Biopsy was avoided in the majority of MRI-negative men (PI-RADS 1-2), while in those who did undergo biopsy within this group, a relatively high proportion was found to have GG≥2 cancer (17%), indicating that these individuals bore a heightened risk based on clinical and laboratory markers ("high risk PI-RADS 1-2"). Note.- Two participants (PI-RADS score 2 and 5) included here revealed GG2 cancer at prostatectomy during the diagnostic phase, but no biopsy was documented. Numbers may not sum to 100% due to rounding.

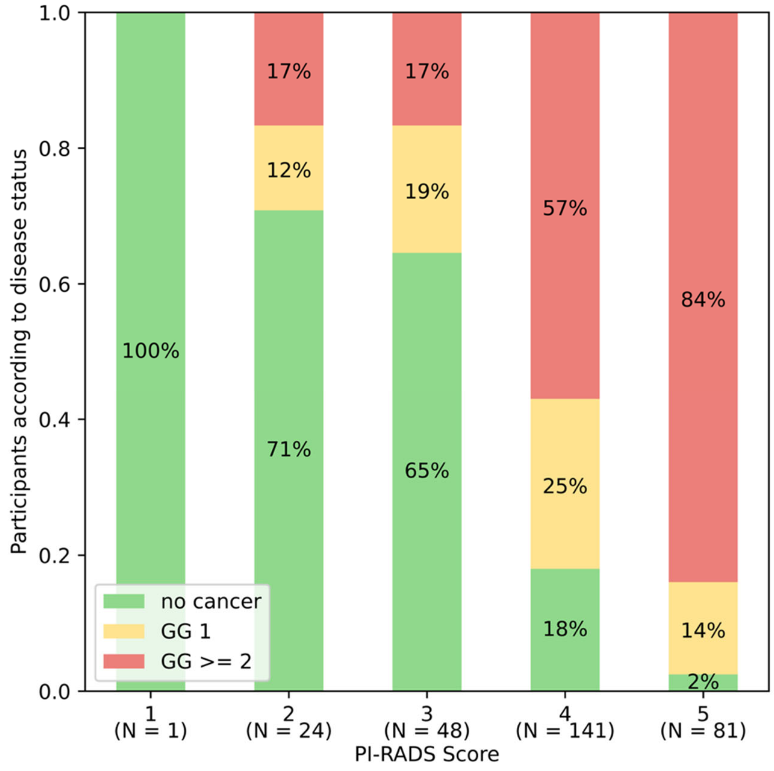

© 2024 Hamm CA et al. *JAMA Oncol.*

eFigure 2. Association between MRI score and histology in ultrasound-guided biopsies and prostatectomy specimens

Men diagnosed with GG≥2 cancer within the first year underwent prostatectomy in 69% (111/161) of cases. 59% of men with GG1 cancer did not undergo prostatectomy (59%, 24/58). Note.- Men with negative MRI and no biopsy or prostatectomy are not included (n=240). PI-RADS= Prostate Imaging–Reporting and Data System; GG= International Society of Urological Pathology grade group.

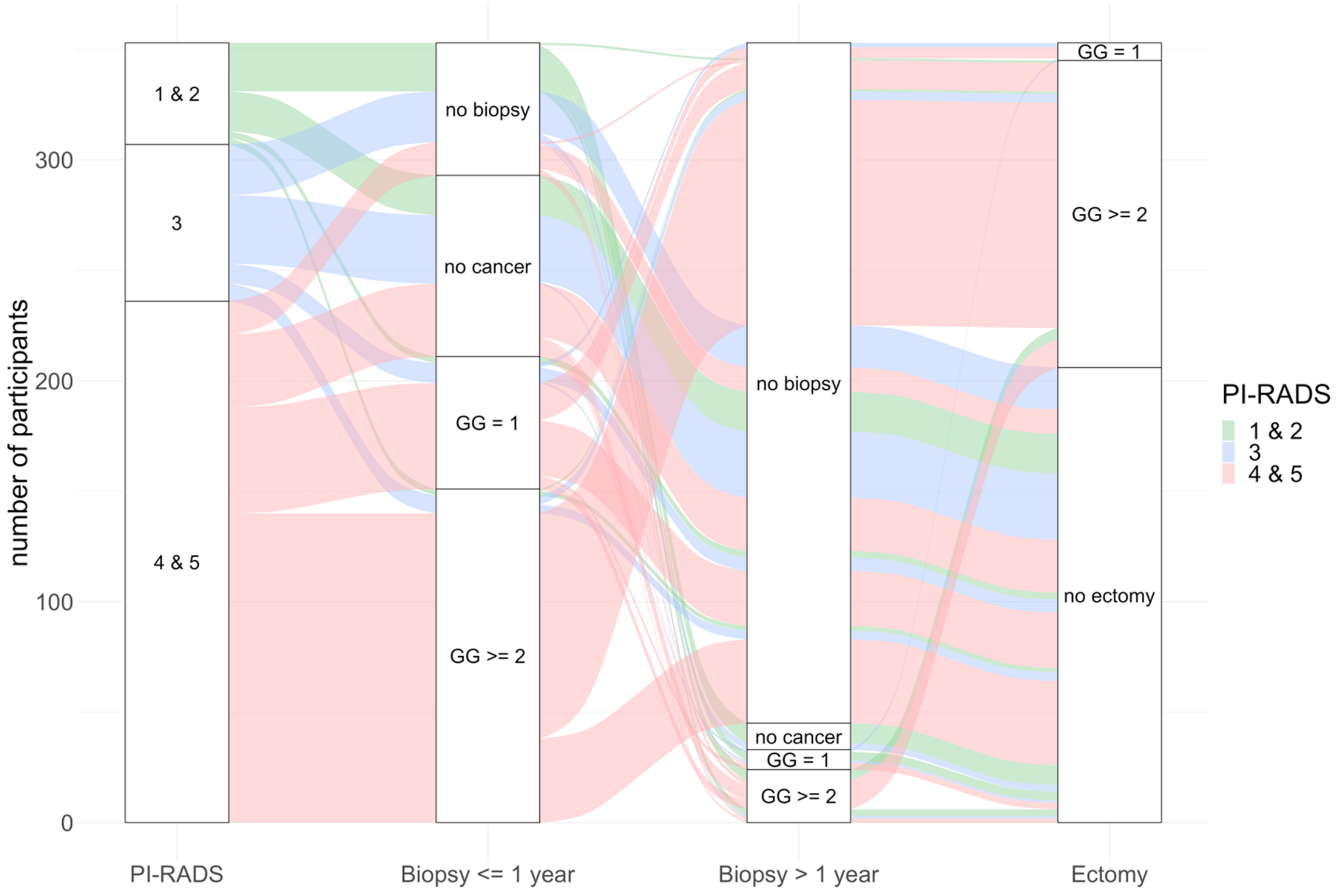

© 2024 Hamm CA et al. *JAMA Oncol.*

eFigure 3. Average time interval between serum PSA tests during active monitoring of MRI-negative men

Note. – The red line indicates the six months interval recommended by the NICE guidelines for serum PSA testing within the safety net.

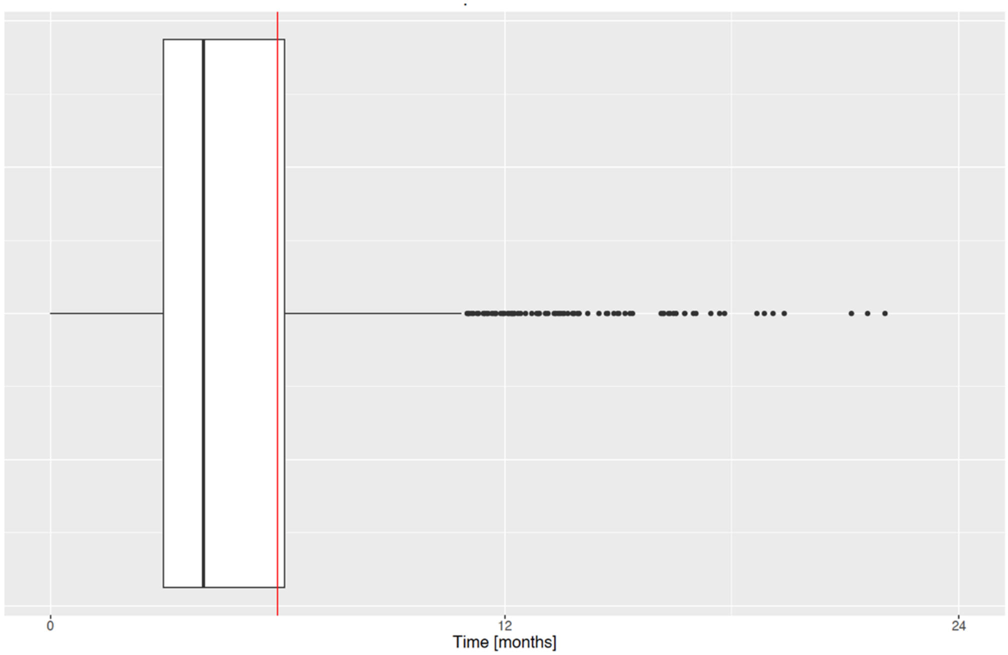

© 2024 Hamm CA et al. *JAMA Oncol.*

eFigure 4. Average time interval between serum PSA tests during active monitoring of MRI-positive men

Note. – The red line indicates the six months interval recommended by the NICE guidelines for serum PSA testing within the safety net of MRI-negative men.

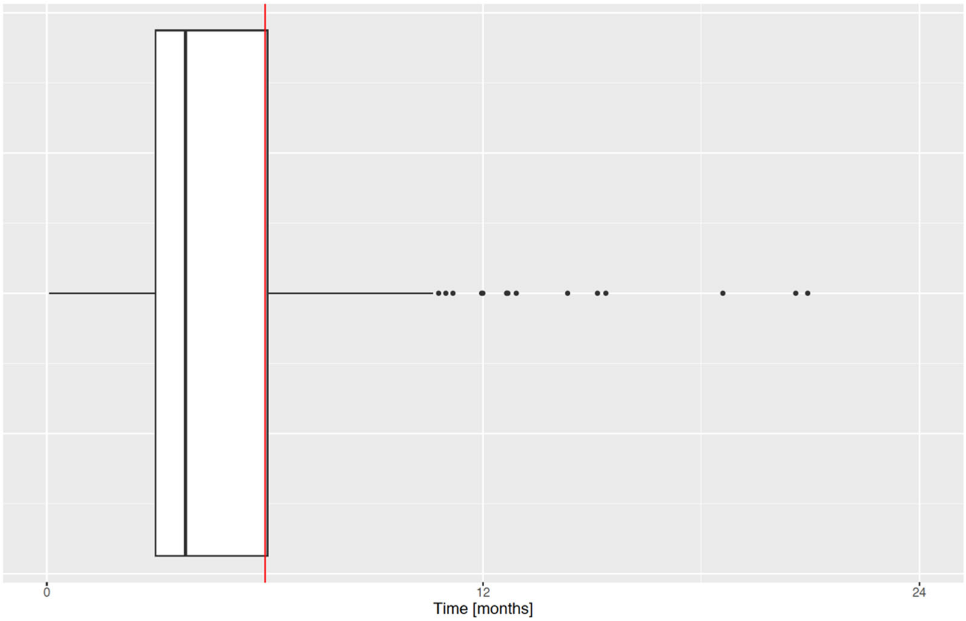

© 2024 Hamm CA et al. *JAMA Oncol.*
