# Supplementary material for: Oncological Safety of MRI-Informed Biopsy Decision-Making in Men With Suspected Prostate Cancer
Source: JAMA Oncol. 2024 Dec 12;11(2):145–53. doi: 10.1001/jamaoncol.2024.5497 (PMC11843366; doi:10.1001/jamaoncol.2024.5497)
Supplement: Supplement 2. — Data Sharing Statement [file jamaoncol-e245497-s002.pdf]

Data Sharing Statement

Hamm CA. Oncological Safety of MRI-Informed Biopsy Decision-Making in Men With Suspected Prostate Cancer. *JAMA Oncol*. Published December 12, 2024. doi:10.1001/jamaoncol.2024.5497

Data
Data available: No

Additional Information
Explanation for why data not available: Due to the sensitive nature of patient data involved in this study and the absence of explicit consent from study participants, study data cannot be made publicly accessible. For any specific inquiries or collaboration requests, please contact the corresponding author.
